# Dementia Is Associated with In-Hospital Mortality and Prolonged Length of Stay: A Propensity Score Matched Analysis on Administrative Data

**DOI:** 10.3390/healthcare13222913

**Published:** 2025-11-14

**Authors:** Giuseppe Di Martino, Pamela Di Giovanni, Federica Vaccaro, Livia Tognaccini, Edoardo Trebbi, Teresa Aita, Ferdinando Romano, Tommaso Staniscia

**Affiliations:** 1 Department of Medicine and Ageing Sciences, “G. d’Annunzio” University of Chieti-Pescara, 66100 Chieti, Italy; pamela.digiovanni@unich.it (P.D.G.); federica.vaccaro@unich.it (F.V.); tommaso.staniscia@unich.it (T.S.); 2 Unit of Epidemiology and Health, Local Health Authority of Pescara, 65100 Pescara, Italy; 3 Department of Public Health and Infectious Diseases, “La Sapienza” University of Rome, 00100 Rome, Italy; livia.tognaccini@uniroma1.it (L.T.); ferdinando.romano@uniroma1.it (F.R.); 4 Local Health Authority of Avezzano-Sulmona-L’Aquila, 67100 L’Aquila, Italy; taita@asl1abruzzo.it

**Keywords:** dementia, epidemiology, mortality, propensity score, health-services research, Italy

## Abstract

**Highlights:**

**What are the main findings?**

- Patients with dementia are hospitalized for different diseases compared to patients without dementia.
- Dementia was associated with in-hospital mortality.
- Dementia was associated with prolonged length of stay.

**What are the implications of the main findings?**

- The definition of the causes of these differences aims to improve surveillance systems.
- It is important to implement better management of dementia during hospital admission.
- Better management of dementia is also important to shorten discharge delay to other healthcare facilities.

**Abstract:**

**Background/Objectives**: To investigate the relationship between dementia and hospital outcomes (in-hospital mortality and prolonged length of stay). **Methods**: A retrospective study was conducted considering all hospital admissions performed between 1st January 2018 and 31st December 2023 in the Abruzzo region, Italy. The study was conducted on a large sample including all elderly patients admitted to hospital in a Southern Italian region during a six year period. To compare outcomes between patients with and without dementia, a propensity score matching procedure was performed using a multivariable logistic model adjusted for age and gender and comorbidities. Odds ratios for primary and secondary outcomes were computed using logistic regression models. **Results**: After the matching procedure, 25,476 patients were included in the analyses: 12,738 with dementia and 12,738 controls. Logistic regression models showed that dementia was associated with in-hospital mortality (OR: 2.02; 95% CI 1.91–2.18; *p* < 0.001) and prolonged length of stay (OR: 1.44; 95% CI 1.29–1.58; *p* < 0.001). **Conclusions**: In a large cohort of Italian patients, dementia was associated with in-hospital mortality and prolonged length of stay.

## 1. Introduction

Dementia is a common disease in geriatric patients, reaching a worldwide prevalence of 697 cases in over 10,000 inhabitants, and it is still rising [1]. The number of subjects living with dementia approximately doubles every five years, particularly among female patients [2]. Subjects with this condition represent a heavy burden for public health, both for clinical and economic issues. Most cases of dementia in reality are not due to a single pathological process, but represent the sum of more pathological processes arising in brains with varying degrees of presentation [3]. In fact, dementia includes a large spectrum of different diseases. Firstly, there is the differentiation in vascular and non-vascular disease. The vast majority of non-vascular diseases were included in six main categories of neurodegenerative proteinopathy: amyloid-β (Aβ), microtubule-associated protein tau, TAR DNA-binding protein 43 (TDP-43), fused in sarcoma (FUS), α-synuclein, and prion protein [4]. Secondly, three clinical categories—early versus late, gradual versus rapid, and sporadic versus familial—were considered separately or in combination to the spectrum of dementing processes [4]. On one hand, dementia causes high costs for primary care, and, on the other hand, it represents a frequent cause of hospital admission [3]. In fact, it has been reported that the great part of the costs for subjects affected by dementia resides in hospital care management [3,4]. Among subjects discharged from general hospitals, the proportion of patients with dementia ranged between 4 and 30% [5,6] of the total amount of hospital admissions. It has been reported that dementia is generally associated with a higher rate of hospitalization, increased length of stay (LOS), and increased in-hospital death [7,8,9]. Some studies in the literature show that the pattern of clinical outcomes and concomitant illness is related to the presence or absence of dementia; in particular, cardiovascular diseases (CVDs), pneumonia, accidents, and cancer are the principal causes of mortality among in-patients with dementia [10]. However, data on the link between comorbidity and in-hospital death among patients with dementia are poor and not deeply investigated. In particular, it is unclear whether worse clinical outcomes are directly linked to dementia-associated morbidity or are primarily related to the comorbid conditions of the patient. In fact, dementia is frequently related to a specific comorbidity pattern [11]. In addition, several studies reported that cognitive impairment and poor mobility at hospitalization are independently associated with poor hospital outcomes and prolonged length of stay (PLOS) [12,13,14,15].

In order to investigate the association between dementia and hospital outcomes, a retrospective study was conducted in a large sample including all elderly patients admitted to hospital in a Southern Italian region during a six year period, evaluating the hospital discharge records (HDRs).

## 2. Materials and Methods

### 2.1. Study Design

It was a retrospective observational study that considered all hospital admissions performed between 1st January 2018 and 31st December 2023 in the Abruzzo region, Italy. Abruzzo is a region of Southern Italy, reporting about 1.3 million inhabitants and 27 hospitals, comprising 16 public hospitals and 11 private clinics [16]. Its healthcare system is organized into four local health authorities (LHAs) according to the province’s organization. Data were extracted from HDRs, including information on the patient’s demographic characteristics, a diagnosis-related group code (DRG) was used to classify the hospitalization, and up to 6 diagnoses and 6 procedures were performed during the hospitalization, coded as per the International Classification of Disease, 9th Revision, Clinical Modification (ICD-9-CM). All hospitalizations related to surgery procedures and delivery were excluded from the analysis and only patients aged over 50 years were included. Subjects with repeated hospitalizations were counted only for the first admission. Among included hospitalizations, patients reporting codes 290.x (all dementia types), 294.1 (dementia in other clinical conditions classified elsewhere), and 331.2 (senile degeneration of the brain) were considered as affected by dementia. In order to evaluate comorbidities, all diagnoses included in the Charlson’s Comorbidity Index (CCI) were identified and extracted, according to the coding algorithm proposed by Quan et al. [17]. Admissions with a duration of over 12 days were considered as ‘prolonged length of stay’ (PLOS). This value represented the upper quartile of the LOS distribution of the overall population included in the study. The study outcomes were in-hospital mortality and PLOS. As a secondary analysis, gender differences in the study outcomes were explored.

### 2.2. Statistical Analyses

Quantitative variables were reported as mean and standard deviation (SD) or median and interquartile range (IQR) according to data distribution. Qualitative variables were reported as frequency and percentage. Annual proportion of dementia admissions was calculated. In order to confront study outcomes between patients with and without dementia, a propensity score matching (PSM) procedure was developed using a multivariable logistic regression model with a 1:1 greedy matching algorithm with a caliper of 0.05. In addition to age and gender, all comorbidities included in the CCI were included in the matching procedure. The adequacy of model covariates’ balance in the matched sample was assessed with standardized mean differences (SMDs) between the two study groups. A SMD lower than 10% was considered as a threshold for the evaluation of good balance between study groups [18]. Patients for whom no match was found were discarded from the matched analyses. After PSM procedure, odds ratios with their 95% confidence intervals (95% CI) for in-hospital mortality and PLOS were computed using logistic regression models adjusted for propensity score as covariate. A 2-tailed *p* value less than 5% was considered as significant. The statistical analysis was developed using Stata Software v20 (Stata Corp. LLC, 4905 Lakeway Drive, College Station, TX, USA).

## 3. Results

During the study period (2018–2023), a total of 987,763 hospital admissions were performed in the Abruzzo region. Among them, 641,035 matched the inclusion criteria. Table 1 reported the characteristics of included patients. Patients with dementia were older than patients without dementia (84.96 vs. 72.04 years) and they were more frequently female (61.55% vs. 47.59%). Dementia patients reported longer LOS (median 9, IQR 5–14) compared to controls (median 6, IQR 2–12). Among comorbidities, patients with dementia more frequently reported chronic heart failure (CHF) (18.57% vs. 10.69%), cerebrovascular diseases (24.42% vs. 9.09%), and diabetes (14.04% vs. 10.15). On the other hand, patients without dementia more frequently reported myocardial infarction (3.04% vs. 1.28%) and cancer (12.18% vs. 4.51%). After the matching procedure, 25,476 patients were included in the analyses: 12,738 with dementia and 12,738 controls.

Among causes of admission, both study groups reported “Pulmonary edema and respiratory distress” as the main cause of admission. Compared to controls, patients with dementia more frequently were admitted for Pulmonary edema (10.55% vs. 4.24%), sepsis (8.89% vs. 2.34%), and hearth failure (6.13% vs. 3.94%). In addition, renal failure (4.15%) represents one of the five most frequent causes oof admissions, compared to controls, who reported lower frequency (9277 cases, 1.48%). Most frequent diagnoses are reported in Table 2.

Among study outcomes, patients with dementia reported a higher in-hospital mortality (2052 cases, 16.11%) compared to controls (33,785 cases, 5.38%). After matching, the proportion of in-hospital mortality among patients without dementia was increased (1108 cases, 8.7%), but still lower than dementia patients. About PLOS, dementia patients reported a higher frequency, with 5178 cases (40.65%) compared to controls (190,637 cases, 30.34%). A similar proportion was observed after matching (4114 cases, 32.30%). Logistic regression analyses showed that dementia was associated with in-hospital mortality (OR: 2.02; 95% CI 1.91–2.18; *p* < 0.001) and prolonged length of stay (OR: 1.44; 95% CI 1.29–1.58; *p* < 0.001), as reported in Table 3.

A supplementary analysis of gender showed that male gender reported higher likelihood of in-hospital mortality (OR: 1.89; 95% CI 1.75–2.04; *p* < 0.001) compared to female (OR: 1.45; 95% CI 1.36–1.55; *p* < 0.001). The same results were reported for PLOS, where males reported higher association (OR: 1.54; 95% CI 1.46–1.64; *p* < 0.001) compared to females (OR: 1.11; 95% CI 1.06–1.16; *p* < 0.001).

In addition, the risk of mortality with dementia decreases with age. In particular, as reported in Table S1, patients aged between 60 and 70 years reported a higher likelihood of in-hospital mortality (OR: 3.87; 95% CI 2.55–5.88; *p* < 0.001) compared to patients aged over 80 years (OR: 1.62; 95% CI 1.53–1.71; *p* < 0.001)

Finally, patients with dementia were more frequently discharged to long-term care facilities (LTCFs) or nursing homes, compared to controls (10.87% vs. 1.83%).

## 4. Discussion

The present study evaluated the relationship between dementia, in-hospital deaths, and PLOS in a large sample of hospital admissions which occurred between the years 2018 and 2023 in a Southern Italian region. The analysis of comorbidities highlighted that dementia patients more frequently reported CHF, COPD, and cerebrovascular diseases compared to patients without dementia. These characteristics were in line with the previously published literature [11]. Additional risk factors for dementia include age, family history of Alzheimer’s disease (AD), hypertension, diabetes, chronic inflammation, traumatic brain injury, and low education [4]. In fact, new epidemiological models suggest that vascular disease and dysregulated inflammation are early risk factors for dementia and AD [4].

About study outcomes, this study confirmed that dementia patients have a significantly higher likelihood of in-hospital death compared to patients without dementia, as stated by several studies based on different methodologies [3,5,6,11].

The real cause of this higher mortality was not deeply evaluated and clarified in the literature; however, it is known that dementia is associated with a frailty condition that can directly impact the study outcome [17]. It is known that patients with dementia are more frequently older and usually report more comorbidities compared to patients without dementia [18], but the PS matching procedure was performed to avoid the confounding effects of baseline characteristics. Also, prospective studies from other countries confirmed these results: Agero-Torres et al. [19] reported a double risk of in-hospital death for patients with dementia compared to the controls. Also, a study from Brazil [20] showed a significant association between delirium and in-hospital death, reporting an OR of over four points. It is important to highlight that no similar study performed used a matching procedure. It is important to highlight how the present study was focused on the hospital environment where dementia patients who reported a usual pattern of comorbidities were usually admitted during the end-stage of their conditions. Patients with start-stage dementia are rarely hospitalized and they are frequently managed at home or in long-term care facilities with positive outcomes. Above all, end-stage dementia requires hospital care because bed-rest syndrome can frequently lead to conditions such as respiratory complications, like ab ingestis pneumonia, and infections. In fact, it is known that 29.69% of subjects with dementia died from pneumonia and its complications [19,21]. The increase in OR by age class, reported in Table S1, can be due to the stronger impact of dementia in younger patients compared to older. Probably, the in-hospital mortality among elderly patients could be referred to other conditions than dementia, where cognitive decline is often a comorbidity and not a cause of death.

About LOS, this study confirmed that dementia was associated with a prolonged hospital stay. Several studies reported similar data [12,22,23]. Several factors can influence the LOS, other than comorbidities: the healthcare system, the presence of neuropsychiatric symptoms, income status of the patient and of their family, and insurance status can all affect the duration of LOS [12]. Frequently, the onset of delirium or agitation during hospitalization can increase the duration of the hospitalization alongside the baseline comorbidities [24].

Also, it is important to highlight how end-stage dementia cannot be easily managed at home, so the discharge process can be dilatated by the availability of beds in LTCFs. The lack of beds in this kind of structure can also prolong the hospital stay. This point, which also frequently represents a cause of PLOS for other conditions [25], is highlighted in this study, with over 10% of patients with dementia discharged to LTCFs instead of home. The knowledge of the presence of dementia as a comorbidity should cause physicians to accelerate the disposal of discharge to LTCFs with appropriate bed-management. Also, LHAs and policymakers should improve the availability of LTCFs in order to reduce the LOS of complex patients such as those with dementia [26].

Another relevant factor that can lead to PLOS was the possible underestimation of dementia prevalence. It is well known that early-stage dementia is frequently undiagnosed, varying between 43% and 58% in Europe [27]. So, hospital admission can trigger newly onset dementia, leading to the development of delirium and agitation [28].

The knowledge of worse outcomes among patients with dementia can be useful for hospital management to implement a multidisciplinary approach. In particular, physicians should focus on the management of patients with dementia in order to better manage the disease and to avoid worse outcomes. In addition, it is suggested to understand the possible discharge modality in order to preemptively organize the transfer to other settings, such as LTCFs.

In addition, the implementation of home-based interventions in primary care integration could help in the management of the condition [29].

### Strengths and Limitations

The major strength of this study is represented by the large population studied, making the study results very robust. No previous study on dementia used PSM, so these results are robust and can be generalized. PSM is also a strong methodology that can limit possible confounders, as shown in previous studies [30]. The use of PSM allows us to correct the influence of comorbidities and patient characteristics, such as gender. In fact, as confirmed by this study, dementia is a female-oriented disease. In parallel, some limitations should be considered. Firstly, the identification of diagnosis is based on ICD-9-CM codes that are not able to consider the severity of each condition. The underestimation of conditions and the lack of information on disease severity could bias the results. In particular, patients with dementia could report worse outcomes in reality compared to the study. Secondly, the use of administrative data may be limited by the lack of important information such as drug therapy, laboratory results, and performance status. However, some diseases included in the CCI, such as MI, COPD, and hemi/paraplegia, are important contributors to poor performance status in older people. Finally, the real prevalence of dementia or other comorbidities could be underestimated, due to underreporting in the HDRs. Administrative data were structured for management and economic purposes and not for epidemiological aims. Specifically, HDRs in Italy were introduced during the 1990s for administrative reasons, allocating a specific tariff to each hospitalization by the calculation of a diagnosis-related group (DRG). Some comorbidities do not influence the DRG, so their addition in the HDR does not change the price of the admission and, for this reason, they are sometimes not reported. However, it is a standardized source of data, largely used in Italy for health system research.

## 5. Conclusions

In a large cohort of Italian patients, dementia was associated with in-hospital death and prolonged length of stay. The definition of the causes of these differences aims to improve surveillance systems and implement better management of this comorbidity during hospital admission.

## Figures and Tables

**Table 1:** Patient’s characteristics.

	Unmatched Population (n = 641,035)		Matched Population (n = 25,476)	Standardized Mean Difference (%)
	No dementia (n = 628,288)	Dementia (n = 12,738)	No dementia (n = 12,738)	
Age *mean* ± *SD*	72.04 ± 11.32	84.96 ± 7.18	83.66 ± 7.32	1.30
Male gender *n (%)*	329,306 (52.41)	4898 (38.45)	4896 (38.40)	0.07
MI *n (%)*	19,085 (3.04)	163 (1.28)	165 (1.29)	0.01
CHF *n (%)*	67,141 (10.69)	2366 (18.57)	2370 (18.59)	0.03
Vascular diseases *n (%)*	18,615 (1.97)	205 (1.61)	204 (1.60)	0.01
Cerebrovascular diseases *n (%)*	57,085 (9.09)	3110 (24.42)	3089 (24.23)	0.17
COPD *n (%)*	37,106 (5.91)	1032 (8.10)	998 (7.83)	0.27
Rheumatic diseases *n (%)*	5771 (0.92)	52 (0.41)	54 (0.42)	0.01
Liver diseases *n (%)*	12,594 (2.00)	185 (1.45)	169 (1.32)	0.13
Diabetes *n (%)*	63,777 (10.15)	1788 (14.04)	1821 (14.28)	0.25
Renal diseases *n (%)*	38,100 (6.06)	1154 (9.06)	1148 (9.00)	0.05
Cancer *n (%)*	76,503 (12.18)	575 (4.51)	581 (4.56)	0.05

Abbreviations: SD = standard deviation; MI = myocardial infarction; CHF = chronic heart failure; COPD = chronic obstructive pulmonary disease.

**Table 2:** Most frequent causes of admission among patients with and without dementia.

Dementia (n = 12,738)	N (%)	No Dementia (n = 628,288)	N (%)
Pulmonary edema and respiratory distress (DRG = 87)	1344 (10.55)	Pulmonary edema and respiratory distress (DRG = 87)	26,617 (4.24)
Sepsis without mechanical ventilation support (DRG = 576)	1133 (8.89)	Heart failure and shock (DRG = 127)	24,774 (3.94)
Heart failure and shock (DRG = 127)	781 (6.13)	Sepsis without mechanical ventilation support (DRG = 576)	14,732 (2.34)
Intracranial hemorrhage or cerebral infarction (DRG = 14)	583 (4.58)	Intracranial hemorrhage or cerebral infarction (DRG = 14)	11,331 (1.80)
Renal failure (DRG = 316)	528 (4.15)	Respiratory infections (DRG = 79)	11,257 (1.79)

Abbreviation: DRG = diagnosis-related group.

**Table 3:** Outcome comparisons between patients with and without dementia.

Outcomes	Dementia + n(%)	Dementia − n(%)	Crude OR (95% CI)	aOR * (95% CI)	*p*-Value
In-hospital mortality	2052 (16.11)	1108 (8.7)	3.38 (3.21–3.55)	2.02 (1.91–2.18)	<0.001
PLOS	5178 (40.65)	4114 (32.30)	1.57(1.52–1.63)	1.44 (1.29–1.58)	<0.001

Abbreviations: OR = odds ratio; aOR = adjusted odds ratio; CI = confidence interval; PLOS = prolonged length of stay. * All models were adjusted for propensity score.

## Data Availability

Data were not available due to privacy restrictions.

## Supplementary Materials

The following supporting information can be downloaded at https://www.mdpi.com/article/10.3390/healthcare13222913/s1. Table S1. Mortality comparison between patients with and without dementia by age categories.
