# Supplementary material for: Dementia Is Associated with In-Hospital Mortality and Prolonged Length of Stay: A Propensity Score Matched Analysis on Administrative Data
Source: Healthcare (Basel). 2025 Nov 14;13(22):2913. doi: 10.3390/healthcare13222913 (PMC12652701; doi:10.3390/healthcare13222913)
Supplement: Supplementary file 1 [file healthcare-13-02913-s001.zip › healthcare-3916896-supplementary.pdf]

Table S1. Mortality comparison between patients with and without dementia by age categories

	Mortality Dementia + n(%)	Mortality Dementia - n(%)	aOR* (95%CI)	p-value
60-69 years	26 (8.28)	293 (2.30)	3.87 (2.55–5.88)	<0.001
70-79 years	243 (11.9)	540 (4.24)	3.08 (2.68–3.54)	<0.001
Over 80 years	1,777 (17.21)	1,462 (11.47)	1.62 (1.53–1.71)	<0.001
